# Patient preferences in allergy immunotherapy (AIT) in Germany – a discrete-choice-experiment

**DOI:** 10.1186/s13561-016-0110-x

**Published:** 2016-08-02

**Authors:** Kathrin Damm, Janina Volk, Andreas Horn, Jean-Pierre Allam, Ninette Troensegaard-Petersen, Niels Serup-Hansen, Thomas Winkler, Ivonne Thiessen, Kathrin Borchert, Eike G. Wüstenberg, Thomas Mittendorf

**Affiliations:** 1 Center for Health Economics Research Hannover (CHERH), Leibniz University Hannover, Hannover, Germany; 2 ALK-Abelló Arzneimittel GmbH, Griegstraße 75, Haus 25, D-22763 Hamburg, Germany; 3 ENT Practice am Neckar Dres. Zeuner, Horn & Vasvari, Heidelberg, Germany; 4 Department of Dermatology and Allergy, University of Bonn, Bonn, Germany; 5 ALK-Abelló, Hørsholm, Denmark; 6 Xcenda GmbH, Hannover, Germany; 7 Department for Otorhinolaryngology, Medical Faculty Dresden University, Dresden, Germany

**Keywords:** Allergic rhinitis, Allergy immunotherapy, Discrete-choice-experiment, Patient preferences, Sublingual immunotherapy-tablet

## Abstract

**Background:**

Allergic Rhinitis (AR) is a common disorder in Europe with Allergic Asthma (AA) as a frequent comorbidity. Allergy immunotherapy (AIT) is the only causal therapy of AR and AA, and can be administered as subcutaneous injections at the physician or as sublingual drops or tablets at home. The usual treatment duration is 3 years.

**Objective:**

This study aimed to elicit patient preferences to identify the AIT administration mode preferred by patients.

**Methods:**

A discrete-choice-experiment (DCE) was developed to determine how people weight different treatment options using a paper-based questionnaire from June to September 2014, including 16 study centres. Main inclusion criteria: >18 years, grass, birch and/or house dust mite AR with moderate to severe symptoms, AIT-naïve and AIT-indicated. DCE-attributes were: Administration form, number and duration of physician visits, frequency of life-threatening anaphylactic shocks, local side-effects and co-payments.

**Results:**

Two-hundred thirty-nine subjects participated, resulting in analysable 1842 choices. All attributes were significant predictors for the treatment-choice. Ranked by importance, the following first three attributes are most preferred by patients:

- 1^st^ Number and duration of physician visits:
  - Fewer visits with shorter duration preferred (0.658*)
- 2^nd^ Frequency of life-threatening anaphylactic shocks:
  - Lower risk of shocks preferred (0.285*)
- 3^rd^ Local side-effects:
  - Preference for rash/swelling on upper arm over itching/swelling under the tongue (0.210*)
  - (*coefficient-size represents relative importance of the attributes)

**Conclusion:**

The most important attribute is the number and duration of visits to a physician. A lower risk of life-threatening anaphylactic shocks was ranked as the second whereas co-payments and administration form play a limited role.

## Background

Allergic rhinitis (AR) is a common disorder in Europe, affecting more than one out of five adults and representing a considerable burden both on the individual patient and the society [1, 2]. AR is associated with comorbidities as well as long-term consequences frequently including allergic asthma (AA). More than one third of allergy patients are also diagnosed with asthma. In addition, AR is associated with burdensome symptoms, a loss of productivity as well as the impairment of daily activities and quality of sleep [3, 4]. Accordingly, in subjects with AR, the health-related quality of life is significantly affected [5].

Allergy immunotherapy (AIT) is a form of treatment that induces a protective immune response and aims to treat the underlying immunological cause of the allergy. Besides of symptom relief, also long-term efficacy and the prevention of other allergic diseases, such as the development of asthma, are intended. AIT results in a significant reduction of the symptom burden and medication need [6].

AIT can be administered by subcutaneous injections (SCIT), followed 30 min monitoring for side effects, at the physician’s office or as sublingual drops or tablets (SLIT-drops or SLIT-tablets) at home [7, 8]. There are products in both administration forms for which, after 3 years of continuous AIT, a sustained treatment effect after treatment stop has been observed [8, 9]. Available AIT not only differs regarding the administration form, the therapy options also differ regarding the type of local side-effects, the risk of a life-threatening anaphylactic shock, co-payments and the number of necessary visits to a physician [6, 10].

An increasing number of patient preference studies are being conducted as measuring patient preferences is a very important component to understand how healthcare in an indication may or should evolve in the future. The aim of preference-based evaluation methods is to assess and weight benefits, costs and risks of therapy options with respect to the individual needs of patients. Analysing the preferences of patients that are intended for an AIT treatment can help to identify distinct features of therapies that have a major influence on the patient benefit. Those insights should be implemented to provide an improved provision of healthcare. The identification of product or service determinants that are preferred by patients is therefore described to enable improved compliance, optimised outcomes and can help to guide drug development [11, 12]. In the current German AIT-guideline that focuses on the management of patients with AR and/or AA, the improvement of compliance is defined as a major task of the future, which is needed to ensure the therapy success and causal efficacy of AIT [8].

Summarising, preference-based evaluation methods help to determine suitable or optimised treatment strategies. They enable the creation of a benefit basket which offers a high fit to the individual needs of patients. Basing medical care on the needs and preferences of patients is also acknowledged to enhance the compliance [12].

Accordingly, the aim of the current study was to elicit patient preferences to identify the most important attributes for patients being indicated for AIT and to determine patient preferences in regard to central distinguishing characteristics of the AIT administration forms SCIT and SLIT-tablet. Therefore a discrete-choice-experiment (DCE) was developed, which is a method that is increasingly used and acknowledged to elicit patient preferences in healthcare research.

## Methods

### Theoretical background of DCEs

In health economics, patient preferences are increasingly being elicited by using conjoint analyses, a method to determine how subjects weight and assess different characteristics (or attributes) of a health technology or service in terms of their importance. Based on the Demand Theory developed by Lancaster in 1966 [13], the preference measurement applied in this approach assumes that a therapy is compounded by one or more different attributes (e.g. visit frequency or side-effects etc.), which in sum represent a given product [12].

The discrete-choice approach is explicitly recommended and acknowledged by regulatory institutions evaluating methods for the measurement of patient preferences to weight and rank single preferences [14]. Accordingly, the present DCE study was developed in accordance with the recommended methodological practice induced by the German Institute for Quality and Efficiency in Health Care, the IQWiG.

DCEs are a choice-based alternative of the conjoint analysis (Random Utility Theory) [15]. In DCEs, product choices are characterised by different attribute levels (e.g. oral medication or injection). The choices are mostly presented in pairwise different hypothetical treatment options and study participants have to decide for one of the options [16]. It is assumed that individuals will opt for those health products/therapy alternatives that promise a higher degree of satisfaction of individual patient needs in regard of their properties [12]. Further details on the method of DCE can be found e.g. in [17–19].

#### Study design

The study was approved by the ethics committee of Hannover Medical School (votum number: 2149–2014), participants were informed and informed consent was obtained. The study population comprised patients meeting the following inclusion criteria:

- Age: minimum of 18 years
- Fluent in German
- AR with moderate to severe symptoms according to the ARIA guideline [7] for a minimum of 2 years against at least one of the following allergens: grass pollen, birch or house dust mite
- Indication for AIT
- No AIT in the history

Patient recruitment was performed Germany-wide by 16 allergy specialist offices in the ambulatory setting. The patients were asked to complete a paper-based questionnaire. The pilot phase was carried out in April 2014 with 21 AR patients to check the practicality, feasibility and methodology of the questionnaire. The main study was conducted between June and September 2014.

The questionnaire covered the four following main areas:

1. General information about AIT (e.g. different types of AIT, efficacy) and the DCE study format
2. The DCE for eliciting preferences
3. Additional questions evaluating the DCE (5-point Likert scales formulated to probe the DCE results within a mix of methods)
4. Questions on socio-demographic characteristics like gender, age, education, employment status

Additionally, allergy specific questions (type of allergy, severity of symptoms in terms of the ARIA criteria) were answered by the respective study physician in a separate form, to control for the medical inclusion criteria.

#### Establishment of relevant product attributes

DCEs are in general confined to the use of only a limited number of attributes to ensure the feasibility for the participants. To identify relevant attributes describing different administration forms of AIT, a review of existing literature was conducted. Results were discussed within an expert workshop with allergists working in the ambulatory and/or in-patient setting. Five attributes were finally identified to describe the constructed hypothetical treatment options. Each of the attributes was defined by two levels designated to clearly distinguish between SCIT and SLIT-tablet treatment (see Table 1). The total number and duration of necessary visits to a physician was added to the choice set of attributes as that domain was shown to be a relevant discriminating factor between SCIT and SLIT in an earlier study by Peter et al. [10]. In addition, also Sondermann et al. identified the time load of an AIT treatment as a central factor for the patient and for the success in regard to the therapy adherence [20]. The frequency of a life-threatening anaphylactic shock as well as the type of non-life-threatening local side-effects was included to the attributes, since these are, besides efficacy, the most common outcome parameters used in clinical AIT trials [6] and in AIT therapy guidelines to evaluate and compare AIT therapies [8, 9]. The total sum of co-payments is another characteristic to distinguish AIT administration options. The included levels of €60 versus €120 were calculated as average values for filled prescriptions in the public pharmacy over the whole treatment duration of 3 years.

**Table 1:** Attributes, Levels and descriptions included in the DCE

Attributes	Characteristics (Levels) SCIT	Characteristics (Levels) SLIT
Administration form	An injection given every visit at the physician’s office	A tablet taken every day at home
Total number and duration of necessary visits to a physician	24 times for 45 min over 3 years	12 times for 15 min over 3 years
Frequency of a life-threatening anaphylactic shock *(An anaphylactic shock is a sudden, serious, life threatening allergic reaction)*	1 in 10,000 patients	1 in 100,000 patients
Local side-effects *(Local side-effects occur in approximately half of the patients during the first months of treatment)*	Rash or swelling sited at the upper arm	Itching or swelling under the tongue
Sum of co-payments for the whole treatment *(Co-payments are paid at the pharmacy when picking up a prescription)*	€60 over 3 years	€120 over 3 years

As essential pre-condition, the efficacy between SCIT and SLIT-tablets was appointed as being equal within the conducted DCE, based on the required guideline standards and a current meta-analysis comparing SCIT and SLIT [21]. This fact was also explained to the participants in the questionnaire before constructed DCE-choice sets were to be answered.

#### Selection of choice sets

Since it was not feasible to include all available attribute combinations (2^5^ = 32) in the questionnaire, a reduced (or factorial) experimental design was used. For this purpose, an orthogonal main effects plan designed by Street et al. [22] was applied, which allows for a reliable evaluation of preferences [23]. Based on the five AIT attributes, eight hypothetical therapy choice sets were generated. To receive optimal pairs, from which the patient had to select one (immunotherapy A or B) the profiles for the second option of the pair were created by using the fold-over method. Thus, maximal dissimilarity between the two options was achieved. A question including an opt-out option has also been included in the Choice set. Table 2 shows one example of the resulting choice sets.

**Table 2:** Exemplary DCE choice set

	Immunotherapy A	Immunotherapy B	
Administration form	A tablet taken every day at home	An injection given every visit at physician’s office	
Total number and duration of necessary visits to a physician	24 times for 45 min over 3 years	12 times for 15 min over 3 years	
Frequency of a life-threatening anaphylactic shock	1 in 10,000 patients	1 in 100,000 patients	
Local side-effects	Itching or swelling under the tongue	Rash or swelling sited at the upper arm	
Sum of co-payments for the whole treatment	€120 over 3 years	€60 over 3 years	
Question 1: Which of the two therapies would you **use**?	❒_A_	❒_B_	❒_None of them_
Question 2: Which of the two therapies do you **prefe**r even if you don’t use it?	❒_A_	❒_B_	

(the displayed Immunotherapies A and B are just two of a number of hypothetical possibilities)

#### Data analyses

In the current study, the calculation of coefficients was performed by conducting a conditional logit model using the maximum likelihood method being considered as the most appropriate methodological approach. Due to the binary attribute levels, a dummy coding was used. Data entry, clean-up and analysis were performed with the software Excel^®^ (2007), SPSS^®^ (version 22) and SAS^®^ (version 9.3).

## Results

### Patient characteristics

A total of 273 participants completed the questionnaire. 34 of these patients did not meet the inclusion criteria in full (age <18 years: *n* = 2; no AR: *n* = 10; AR, but with only mild symptoms: *n* = 22) and had to be excluded from the analysis, resulting in 239 included participants.

Table 3 shows socio-demographic and disease-specific characteristics of the study participants. 58 % of the included participants were female; most participants were younger than 46 years (70 %) corresponding to a mean age of 38 years. Nearly 60 % had a higher education (university entrance diploma or university diploma). Twenty-five percent of the participants had children (<14 years) that also live in their household. A majority (78 %) of the patients were currently employed, mainly full-time (35–40 h per week). The data regarding the types of allergy shows that a majority of participants suffer from more than one allergy. The cohort seems to offer an expected mix of patients with AR and/or AA with a somewhat higher educational background compared with the general population, which is not unusual in studies of this type.

**Table 3:** Socio-demographic and disease characteristics of study participants

Gender			Number	Percentage
*Valid*			239	100 %
	Male		101	42 %
	Female		138	58 %
Age (yrs.)				
*Valid*			239	100 %
	18–30		88	37 %
	31–45		77	32 %
	46–60		53	22 %
	>60		21	9 %
Education				
*Valid*			233	100 %
	Lower or general education		96	41 %
	Higher education		137	59 %
Children <14 years in household				
*Valid*			229	100 %
	No		171	75 %
	Yes		58	25 %
Currently employed				
*Valid*			234	100 %
	No		51	22 %
	Yes		183	78 %
		Full-time	138	75 %
		Part-time	36	20 %
		Other	6	3 %
		Missing	3	2 %
Type of allergy				
*Valid*			239	100 %
	Birch (of those birch only)		126 (37)	53 % (29 %)
	Grass pollen (of those grass pollen only)		158 (50)	66 % (32 %)
	House dust mite (of those house dust mite only)		91 (33)	38 % (36 %)
Severity of nasal symptoms (assessed by patient)				
*Valid*			229	100 %
	VAS 0–49		30	13 %
	VAS 50–100		199	87 %
	VAS average			72
Severity of symptoms affecting the eyes (assessed by patient)				
*Valid*			227	100 %
	VAS 0–49		75	33 %
	VAS 50–100		152	67 %
	VAS average			57

By using a Visual Analogue Scale (VAS) ranging from 0 (not at all burdensome) to 100 (extremely burdensome), patients were asked to assess the severity of nasal symptoms as well as symptoms affecting the eyes. Table 3 shows that a majority of patients rated their symptoms in the top half of the scale (50–100). The results support the diagnosis given by the study physicians, which means that the cohort is representative of a population with moderate to severe AR.

### Complexity of questionnaire

To evaluate the DCE, the participants were asked to assess the difficulty of the experiment on a 5-point Likert scale ranging from “not at all difficult” to “very difficult”. Most participants (84 %) stated that the questionnaire was “not very”or “not at all” difficult. Only 8 % of the participants assessed it as “somewhat” or “very” difficult. This finding is important as it implies that participants were able to understand the task and were able to express their preferences rather than introducing bias by an inability to understand the questionnaire.

### Preferences in the DCE

One-thousand eight hundred forty-two (or 96 %) out of maximum 1912 choices (239 patients × 8 decisions) were included in the model. 70 choices (4 %) were not included due to missing data, as some of the respondents did not rate all pairs. In nearly 90 % of all cases, the participants would use one of the given treatment options and therefore did not select the provided opt-out option.

First of all, the results show that all attributes included in the model had a significant impact on the choices made by the participants (see Table 4). The model itself is highly significant meaning that the null hypothesis of no relationship between choice and the attributes can be rejected. Hence, all attributes preferences are significant predictors for the choice of a treatment, but with a different magnitude.

**Table 4:** Results of the DCE model (Ranked by importance)

Attributes	Coefficient	Standard error	ChiSq^a^	Pr > ChiSq (significance)
Total number and duration of necessary visits to a physician				
24 times for 45 min over 3 years	−.65838	.05034	171.0415	***(<.0001)
12 times for 15 min over 3 years	0			
Frequency of a life-threatening anaphylactic shock				
1 in 10,000 patients	−.28455	.05032	31.9785	***(<.0001)
1 in 100,000 patients	0			
Local side-effects				
Rush or swelling sited at the upper arm	.21045	.05032	17.4935	***(<.0001)
Itching or swelling under the tongue	0			
Sum of co-payments for the whole treatment				
60 Euros over 3 years	.12593	.05031	6.2647	**(.0123)
120 Euros over 3 years	0			
Administration form				
Injection	.10769	0.04980	4.6771	*(.0306)
Tablet	0			

Significance levels denoted by

*** Highly significant (*p* ≤ 0.001); ** Very significant (*p* ≤ 0.01); * Significant (*p* ≤ 0.05)

^a^ chi-square

### Patient preferences ranked by importance

The levels of the coefficients explain the relative importance of the various attributes. The most important attribute for the decision of the participants is the “Total number and duration of necessary visits to a physician”: Patients preferred less and shorter visits with the highest preference or coefficient value (−0.65838). Participants were significantly less likely to choose an AIT with “24 times for 45 min over 3 years” compared with “12 times for 15 min over 3 years”. The coefficient is negative as “24 times for 45 min over 3 years” was selected as the reference categories and participants decided to opt against this level of the attribute “Total number and duration of necessary visits to a physician”.

The second and third most important attributes in the ranking are the “Frequency of a life-threatening anaphylactic shock” (coefficient: −0.28455) and the type of “Local side-effects” (coefficient: 0.21045). Participants were less likely to choose an AIT with a higher frequency of an anaphylactic shock (1 in 10,000) compared with a lower frequency (1 in 100,000) and preferred an AIT that causes “Rash or swelling sited at the upper arm” compared with “Itching or swelling under the tongue”.

The first three attributes had the highest magnitude in their effects. Less important attributes have been the co-payments and administration form. Participants preferred an AIT with lower over higher co-payments. This attribute, however, had the second lowest importance (0.12593) and is less significant (*p* = 0.0123) compared with the above mentioned.

The attribute “Administration form” achieved the lowest coefficient and lowest significance (0.10769; *p* = 0.0306) and is of less importance from a patient preference perspective compared with the other attributes. As mentioned, 5-point Likert scale questions were formulated to probe the DCE results within a mix of methods. The Likert scale results showed coherence for all preferences except for the administration form, where more patients preferred a tablet over an injection with 49 % preferring the tablet, 32 % an injection and 18 % being neutral.

**Fig. 1:**
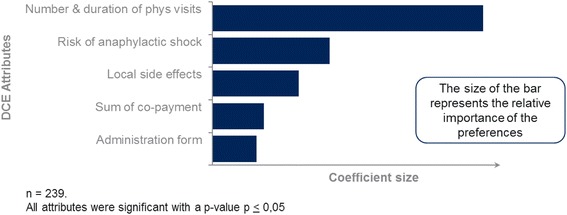
Overview of preference elicitations

Figure 1 shows that the allocated importance, looking at the overall magnitude of the preference expression, was given to those attributes that are in favour of the SLIT-tablet.

Table 4 shows the results (incl. coefficients and significance levels) of all attributes included in the DCE model.

## Discussion

Different estimation methods of DCE data can be found in the literature [17]. In most cases, probit or logit estimations are used. For the current study, the conditional logit model has been evaluated as the best methodological approach. By the use of the DCE method, study participants were implicitly forced to weight different product attributes simultaneously. Hereby, the weaknesses of single-attributive approaches (e.g. Likert scales) with ceiling effects and “all-is-important” results, when asking individuals single disconnected questions, were avoided [24]. Accordingly, the discrete-choice method is explicitly recommended by regulatory institutions evaluating health technologies for the measurement of patient preferences for purposes of weighing and ranking of single preferences [14].

The DCE results of the study showed that allergy patients suffering from AR symptoms have the strongest preference for AIT with less and shorter visits to a physician. Compared with all other attributes included in the DCE, the Total number and duration of necessary visits to a physician had the highest impact on the AIT choice. The effect size of this attribute was rated by the participants as approximately six times as important as the attribute with the lowest preference. This preference for shorter and fewer visits to a physician might be a reason why the demand for AIT products with only few dosages is growing, even if the scientific evidence for clinical efficacy is poor or even missing in most of these products [8]. Looking at the evidence level in terms of efficacy of the existing products in the market, the SLIT-tablet is the only administration form which fulfils both conditions—a low number and duration of visits to a physician [10] and a high level of evidence for efficacy at the same time [8]. This could also be intuitively assumed a reason why especially patients who are (highly) engaged in private and/or working life might be a subpopulation of patients for which an AIT therapy with the SLIT-tablet could fit best.

In AIT, local reactions at the application site as well as systemic reactions can occur. SLIT in general has a lower risk for potentially life-threatening systemic reactions than SCIT, even if SCIT is also considered as a well-tolerated treatment option for allergic diseases in an allergist’s setting [7, 8]. Further, the attribute with the second highest impact on AIT treatment choices was the frequency of a life-threatening anaphylactic shock and the effect size can be rated as approximately half as important as the first attribute. The DCE results show that the included patients had a stable preference for an AIT with a lower probability of experiencing an anaphylactic shock, which is an intuitive finding. But the ranking compared with the other attributes shows that the risk profile of a therapy alternative is of a relatively high importance from the patient perspective. The type of local side-effects was another important AIT attribute. Study participants preferred a “Rash or swelling sited at the upper arm” over “Itching or swelling under the tongue” in the DCE. One reason for this result might be that patients with respiratory allergy are especially sensitive to effects or restrictions in the mouth. Overall, it can be said, also in accordance with the current German AIT guideline, that the SLIT-tablet risk profile is described to be superior compared with the SCIT risk profile [8].

In the German healthcare system, co-payments by the patients for their medication are required. These co-payments are related to the costs of the medications [25]. The co-payments had a minor and slightly less significant impact on the AIT treatment choices in the DCE. As expected, the participants preferred lower compared with higher co-payments, but the relative preference importance of this attribute compared with the other attributes is only small. This might be due to the fact that the participants were willing to pay a higher amount of money for a therapy that matches their preferences. An additional factor might also be that the average educational background was higher than in the normal population. And as a higher educational background is known to be associated with a higher income [26], this correlation might also lead to a lower sensitivity in regard of costs. However, further investigation on willingness to pay by study subjects under different scenarios might offer a deeper understanding of influencing factors in this respect.

The attribute administration form achieved the lowest coefficient, and is of relative less importance from a patient preference perspective. Therefore, it can be concluded, that the attributes frequency of visits and life-threatening anaphylactic shocks were the most important from a patient perspective. Those attributes are clearly expressing the preference in favour of the SLIT-tablet, and when asking patients directly in context of a Likert scale, the results show that the administration form is of major importance to them, and that they prefer a tablet over injections.

Some limitations of the study need to be addressed. A general critique concerning the DCE method (and all other stated preference methods) is that it may not predict real behaviours and choices. However, especially the DCE method emulates consumer behaviour and well-known decision situations [14].

DCEs can only elicit the relative relevance of the attributes that were included in advance. As already stated, the efficacy between SCIT and SLIT-tablet AIT was set equal for this study in the adult therapy setting, as no generalisable distinction between administration forms is indicated [8, 21]. Accordingly, there is no signal from the current study data on the relative importance of the efficacy as an attribute of AIT compared with the other included attributes. As AIT is defined as the only causal therapy of allergic diseases, the AIT-efficacy level, including long-term effects or the prevention of new sensitisations, differs from plain symptomatic treatment. In this sense, the choice of AIT-products should always be based on a decision for the product with the highest documented evidence level to ensure the best possible treatment effect. If two or more different evidence-based treatment options are available, patient preferences should be taken into account.

## Conclusion

As a first major insight of the conducted study, it can be ascertained that there are preferences for existing AIT administration forms in place. Patient preferences play an increasing role in healthcare environment and shall be taken into account where different comparable treatment options are available. Under the precondition of the study that products do have a comparable efficacy and evidence level, the major preference was assigned to the total number and duration of necessary visits to a physician. Co-payments and the administration of a product seem to play a comparably limited role. Looking at currently available AIT treatment options on the market, it can be concluded that SLIT-tablets seems to be the therapy option which presently matches best the preferences of patients considering the efficacy and evidence of available treatment options.

## Key messages

- The study ascertained that there are preferences for existing AIT administration forms in place.
- The most important attribute is the number and duration of visits to a physician, whereby fewer visits with shorter duration were preferred.
- Patient preferences play an increasing role in healthcare environment and shall be taken into account where different comparable treatment options are available.

## Capsule summary

Patient preferences in Allergy Immunotherapy were ascertained by a discrete-choice-experiment, which is increasingly used and acknowledged in health care research. Elicited preferences shall be considered in clinical practice where different comparable treatment options are available.

## Abbreviations

AA: Allergic asthma
AIT: Allergy immunotherapy
AR: Allergic rhinitis
ARIA: Allergic rhinitis and its impact on asthma
DCE: Discrete-choice-experiment
IQWiG: German Institute for Quality and Efficiency in Health Care
N / n: Population
SCIT: Subcutaneous immunotherapy/Injections
SLIT: Sublingual immunotherapy
VAS: Visual analogue scale

## References

[1] Bauchau V, Durham SR (2004). Prevalence and rate of diagnosis of allergic rhinitis in Europe. Eur Respir J.

[2] Bousquet J, Van Cauwenberge P, Khaltaev N, Aria Workshop Group, World Health Organization (2001). Allergic rhinitis and its impact on asthma. J Allergy Clin Immunol.

[3] Crystal-Peters J, Crown WH, Goetzel RZ, Schutt DC (2000). The cost of productivity losses associated with allergic rhinitis. Am J Manag Care.

[4] Spector SL (1997). Overview of comorbid associations of allergic rhinitis. J Allergy Clin Immunol.

[5] Leynaert B, Neukirch C, Liard R, Bousquet J, Neukirch F (2000). Quality of life in allergic rhinitis and asthma. A population-based study of young adults. Am J Respir Crit Care Med.

[6] Hagen A, Gorenoi V, Schönermark MP (2010). Specific immunotherapy (SIT) in the treatment of allergic rhinitis. GMS Health Technol Assess.

[7] Bousquet J, Khaltaev N, Cruz AA, Denburg J, Fokkens WJ (2008). Allergic Rhinitis and its Impact on Asthma (ARIA) 2008 Update. Allergy.

[8] Pfaar O, Bachert C, Bufe A, Buhl R, Ebner C (2014). Guideline on allergen-specific immunotherapy in IgE-mediated allergic diseases. Allergo J Int..

[9] Kleine-Tebbe J, Bufe A, Ebner C, Eigenmann P, Friedrichs F, et al. Specific Immunotherpy (hyposensitization) for IgE-mediated allergic diseases—Guideline of the German Society of Allergy and Clinical Immunology. Allergologie. 2010;33(1):3–34.

[10] Peter RU, Weimer M, Goertzen W, Holdt C, Schreder CH (2012). Zeitaufwand der Hyposensibilisierungstherapie mit subkutaner und sublingualer Applikation unter Praxisbedingungen. Allergologie.

[11] Mühlbacher A, Bethge S, Tockhorn A (2013). Präferenzmessung im Gesundheitswesen: Grundlagen von Discrete-Choice-Experimenten [Measuring Preferences in Healthcare: Introduction to Discrete-Choice Experiments]. Gesundheitsökonomie & Qualitätsmanagement; Stuttgart.

[12] Mühlbacher AC, Bethge S, Ekert S, Tockhorn A, Nübling M (2008). Der Wert von Innovationen im Gesundheitswesen: Spielen die Patientenpräferenzen eine Rolle. RPG.

[13] Lancaster KJ (1966). A new approach to consumer theory. J Polit Econ.

[14] German Institute for Quality and Efficiency in Health Care (IQWiG). Determine patient preferences by means of Conjoint Analysis. https://www.iqwig.de/en/press/press-releases/press-releases/determine-patient-preferences-by-means-of-conjoint-analysis.6227.html. Accessed 29 July 2014.

[15] Mcfadden D, Zarembka P (1974). Conditional Logit Analysis of Qualitative Choice Behavior. Frontiers in Econometrics.

[16] Ben-Akiva ME, Lerman SR (1985). Discrete choice analysis: theory and application to travel demand.

[17] de Bekker Grob EW, Ryan M, Gerard K (2010). Discrete choice experiments in health economics: a review of the literature. Health Econ.

[18] Clark MD, Determann D, Petrou S, Moro D, de Bekker-Grob EW (2014). Discrete choice experiments in health economics: a review of the literature. Pharmacoeconomics.

[19] Louviere J, Hensher D, Swait J (2000). Stated choice methods: analysis and applications. Cambridge Univ.

[20] Sondermann N, Shah-Hosseini K, Henkel K, Schwalfenberg A, Mösges R (2011). Erfolgsfaktoren der Adherence bei Hyposensibilisierung. Allergologie.

[21] Nelson H, Cartier S, Allen-Ramey F, Lawton S, Calderon MA (2015). Network Meta-analysis Shows Commercialized Subcutaneous and Sublingual Grass Products Have Comparable Efficacy. J Allergy Clin Immunol Pract.

[22] Street D, Burgess L, Louviere JJ (2005). Quick and easy choice sets: Constructing optimal and nearly optimal stated choice experiments. Int J Res Mark.

[23] Street DJ, Burgess L (2007). The construction of optimal stated choice experiments, theory and methods.

[24] Mühlbacher AC, Lincke HJ, Nübling M (2008). Evaluating patients’ preferences for multiple myeloma therapy, a Discrete-Choice-Experiment. GMS Psychosoc Med.

[25] Social Code Book V. http://www.sozialgesetzbuch-sgb.de/sgbv/61.html. Accessed 15 Aug 2014.

[26] Autorengruppe Bildungsberichterstattung. Bildung in Deutschland 2012—Ein indikatorengestützter Bericht mit einer Analyse zur kulturellen Bildung im Lebenslauf; W. Bielefeld: Bertelsmann Verlag; 2012.

